# Association of VO₂max With Postoperative Respiratory Complications in Patients Undergoing Lung Resection Surgery: An Exploratory Study

**DOI:** 10.7759/cureus.114377

**Published:** 2026-08-11

**Authors:** Gokul Govindasamy, Vinod Kumar Saka, Sreevathsa Prasad, Sandhiya Selvarajan, Pradeep S., Vishnukanth Govindaraj

**Affiliations:** 1 Pulmonary Medicine, Jawaharlal Institute of Postgraduate Medical Education and Research (JIPMER), Puducherry, IND; 2 Cardiac Surgery, Jawaharlal Institute of Postgraduate Medical Education and Research (JIPMER), Puducherry, IND; 3 Clinical Pharmacology, Jawaharlal Institute of Postgraduate Medical Education and Research (JIPMER), Puducherry, IND; 4 Surgical Oncology, Jawaharlal Institute of Postgraduate Medical Education and Research (JIPMER), Puducherry, IND

**Keywords:** cardio-pulmonary exercise testing, early postoperative complications, lung resection, maximal aerobic capacity (vo2max), post-operative mortality

## Abstract

Introduction: Preoperative risk assessment is important in patients undergoing lung resection, as postoperative respiratory complications and early mortality remain significant concerns. Cardiopulmonary exercise testing provides an integrated assessment of functional capacity, and maximum oxygen consumption (VO₂max) reflects overall cardiopulmonary reserve. It may therefore help identify patients at increased perioperative risk.

Objective: The primary aim of the study was to predict early postoperative respiratory complications using preoperative VO₂max in subjects undergoing lung resection. The secondary aim was to assess 90-day postoperative mortality.

Methodology: This prospective observational study was conducted in the department of pulmonary medicine, in collaboration with the department of cardiothoracic and vascular surgery and surgical oncology at a tertiary care center from January 2024 to November 2025. A total of 46 subjects were included for evaluation for lung resection. All subjects underwent preoperative spirometry and cardiopulmonary exercise testing. Postoperative respiratory complications were assessed during the early postoperative period, and mortality was recorded till 90 days.

Results: The median preoperative VO₂max was 8.91 mL/kg/minute in the postoperative complication group compared with 16.34 mL/kg/minute in subjects without complications (*P* = 0.002). Postoperative respiratory complications (pneumonia with respiratory failure and pleural effusion) occurred in 4 (8.7%) of the 46 subjects. The 90-day postoperative mortality was 3 (6.5%) of the 46 subjects. Subjects who died had a median VO₂max of 7.8 mL/kg/minute, whereas survivors had a median value of 16.21 mL/kg/minute (*P* = 0.007).

Conclusions: Lower preoperative VO₂max was observed in subjects who developed early (within 14 days) postoperative respiratory complications and in those who died within 90 days after lung resection. These findings suggest that preoperative VO₂max may help predict postoperative respiratory complications and mortality within 90 days.

## Introduction

Lung resection remains the definitive treatment for many patients with resectable lung malignancies and is also performed for selected benign pulmonary disorders, including bronchiectasis, aspergilloma, and other localized, destructive lung diseases. Advances in surgical techniques, anesthetic management, and perioperative care have expanded the eligibility for lung resection, allowing surgery to be offered to patients who were previously considered high risk [1].

A substantial proportion of patients undergoing lung resection are elderly smokers with underlying chronic respiratory diseases, particularly chronic obstructive pulmonary disease (COPD). Although perioperative mortality has decreased considerably over the years, postoperative pulmonary complications continue to represent a major cause of morbidity and mortality after lung resection. Respiratory failure, pneumonia, prolonged ventilatory support, and other cardiopulmonary complications contribute to increased healthcare utilization, prolonged hospitalization, and poorer long-term outcomes [1,2].

Traditional preoperative assessment relies on clinical evaluation, pulmonary function testing, and radiological assessment. However, resting measurements may not adequately reflect the integrated physiological reserve required to tolerate major thoracic surgery. As a result, patients with apparently acceptable lung function may still develop significant postoperative complications, while some patients with impaired pulmonary function may successfully tolerate resection [1,3]

Cardiopulmonary exercise testing (CPET) has emerged as an important tool for evaluating functional capacity and cardiopulmonary reserve. By assessing oxygen uptake during exercise, CPET provides a dynamic evaluation of the interaction between the respiratory, cardiovascular, and musculoskeletal systems. Among CPET-derived variables, maximal oxygen consumption (VO₂max) is considered a reliable indicator of overall physiological reserve and exercise capacity. Reduced VO₂max has been associated with an increased risk of postoperative complications, poorer survival, and adverse outcomes following lung resection [4].

Despite increasing recognition of the role of VO₂max in preoperative risk stratification, data evaluating its association with postoperative respiratory complications remain limited in the Indian population. Therefore, the present study was undertaken to assess the association between preoperative VO₂max measured by CPET and postoperative respiratory complications in patients undergoing lung resection surgery.

This study aims to assess the role of CPET and VO₂max as tools for predicting postoperative outcomes in patients undergoing lung resection.

## Materials and methods

Methodology

This was a descriptive longitudinal study done at the Department of Pulmonary Medicine, Jawaharlal Institute of Postgraduate Medical Education and Research (JIPMER), a tertiary care institute in South India, during the period from February 2024 to November 2025. The inclusion criteria were patients above 18 years of age undergoing lung resection surgeries for lung infections, massive hemoptysis, malignancies, and developmental anomalies.

The exclusion criteria were Forced Expiratory Volume in 1 second (FEV1) less than 0.8 L, patients not giving consent, patients unfit for surgery due to non-respiratory causes, patients with contraindications to spirometry and diffusing capacity of the lung for carbon monoxide (DLCO), and patients with contraindications to CPET. Complications such as wound infections, empyema, prolonged hospital stay, air leak, and bronchopleural fistula were considered surgery-related complications and were not included in this study.

Sampling technique: Convenient sampling

Sample Size Calculation

After reviewing the available hospital records and anticipating the proportion of patients post lung resection having respiratory complications as 50% with 15% absolute precision at 5% level of significance, the initial estimate was 43. The corrected estimate was 45 (assuming 5% attrition rate). Institutional Ethics Committee approval was obtained on February 15, 2024, with project number JIP/IEC-OS/2024/02.

Study Procedure

After enrolling in the study, subjects underwent a thorough clinical evaluation and routine preoperative investigations. They were then asked to perform preoperative spirometry and DLCO. Spirometry was interpreted based on the joint technical guidelines of the American Thoracic Society and the European Respiratory Society. DLCO was performed using the single-breath method.

After a gap of 20 minutes, they further underwent CPET. CPET was conducted using a cycle ergometer with the COSMED system (COSMED, Rome, Italy). The following parameters were assessed: aerobic capacity (VO₂), respiratory exchange ratio, anaerobic threshold (AT), maximum heart rate, oxygen pulse, and oxygen saturation. The impact of comorbidities was assessed using the Charlson Comorbidity Index.

After CPET and DLCO, patients underwent surgical intervention. Patients were monitored for the development of postoperative respiratory complications during the in-hospital postoperative period, up to a maximum of 14 days following lung resection surgery. Once-daily postoperative clinical assessment, including vital signs monitoring and symptom inquiry, was performed by the study investigator. Further investigations were undertaken when respiratory symptoms or clinical deterioration were noted. Post-discharge telephonic follow-up was conducted on the 30th and 90th postoperative days.

Complications assessed during the hospital stay included delayed extubation (>12 hours) or re-intubation, atelectasis assessed using chest radiography, pneumonia, pleural effusion assessed using chest radiography, and respiratory failure assessed using arterial blood gas analysis and the need for oxygen supplementation or an increase in oxygen requirement.

Complications such as wound infections, empyema, prolonged hospital stay, air leak, and bronchopleural fistula were considered surgery-related complications and were not included in this study.

*Quality of Life* 

**Figure 1:**
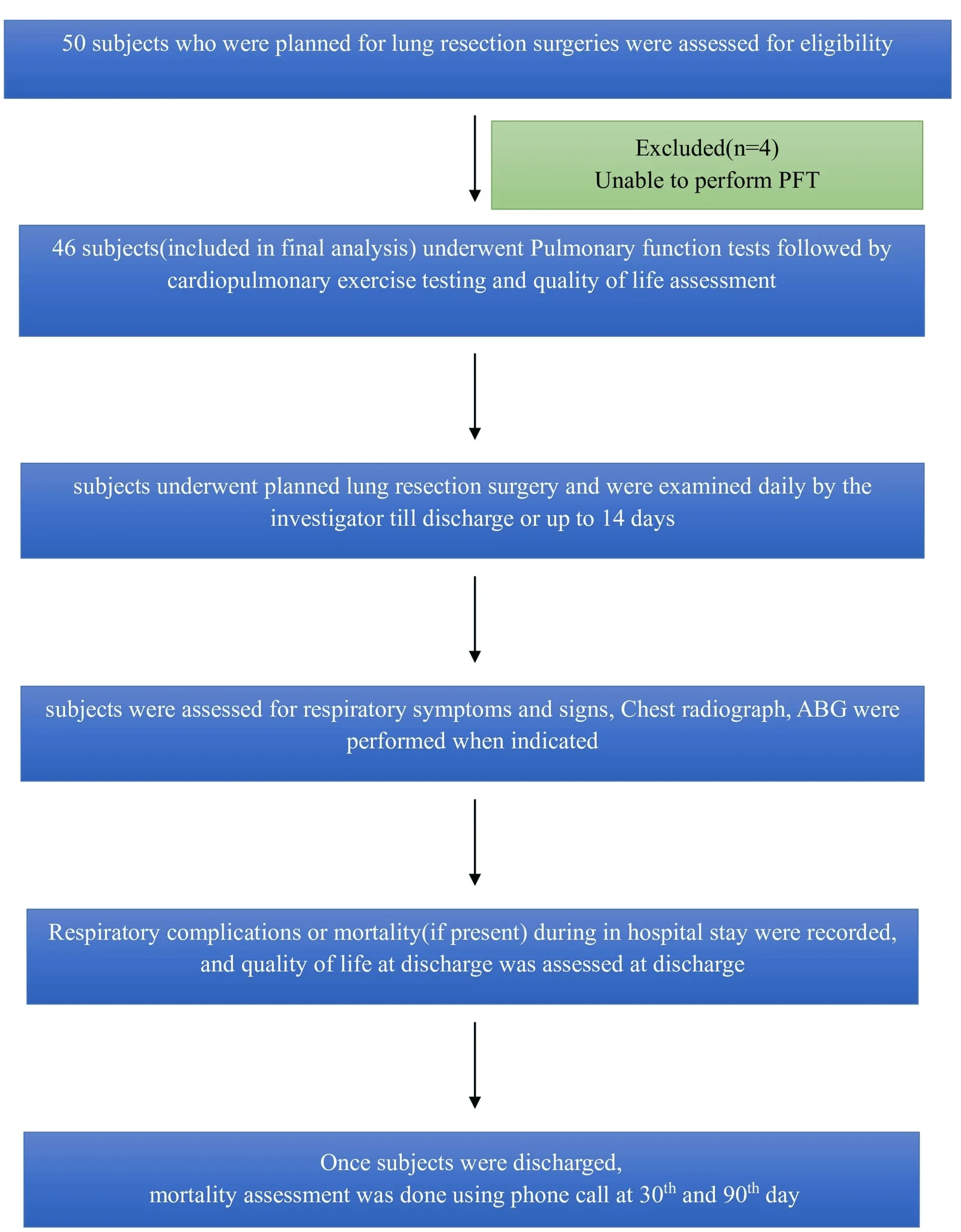
Participant enrollment, study procedure, and follow-up. Image created by the author using Microsoft Word (Microsoft Corporation, Redmond, WA). PFT, pulmonary function test

Assessment of QoL was performed using the EuroQol 5-Dimension (EQ-5D) descriptive system. Subjects completed this questionnaire preoperatively and at the time of discharge. Permission was obtained for use of the questionnaire. Mortality was assessed as 90-day mortality. The brief methodology is shown in Figure 1.

## Results

The present study was conducted in the Department of Pulmonary Medicine in collaboration with the Cardiothoracic and Vascular Surgery and Surgical Oncology departments at a tertiary care center from February 2024 to November 2025.

During the study period, 68 subjects were screened as per a prestructured proforma after obtaining informed written consent. As per the inclusion criteria, 50 patients were included in the study. Among the 50 subjects, 4 were excluded (2 were unable to perform CPET and 2 were unable to perform spirometry). Therefore, a total of 46 subjects were included in the study (Figure 1), and the observations were analyzed.

The mean ± SD age of the study subjects was 46.67 ± 14 years, with the majority of the study subjects, 28 (60.9%), being males. The mean ± SD Body Mass Index (BMI) was 20.76 ± 2.74. All 46 subjects underwent spirometry, DLCO, and CPET. The most common indication for lung resection was malignancy, which constituted 39%. Resection for bronchiectasis constituted the maximum among nonmalignant causes (17.4%). Diabetes and systemic hypertension were the most common comorbidities (15%) observed in the study population. The baseline characteristics of the study population, including the distribution of comorbidities and indications for surgery, are depicted in Table 1. 

**Table 1:** Baseline characteristics of the study population (n = 46). BMI, body mass index; FEV1, forced expiratory volume in one second; DLCO, diffusing capacity of the lung for carbon monoxide; VO₂max, peak oxygen uptake; DM, diabetes mellitus; HTN, hypertension; CAD, coronary artery disease; CLD, chronic lung disease; COPD, chronic obstructive pulmonary disease

Variable	Value
Age (years)	46.67 ± 14.00
BMI (kg/m²)	20.76 ± 2.74
FEV₁ (mean ± SD) L	2.02 ± 0.56
DLCO mmol/min/kPa (mean ± SD)	5.04 ± 1.17
Maximum heart rate (mean ± SD)	166.3 ± 15.06
VO₂max, median (IQR)	16.08 (14.98-17.20)
Anaerobic threshold, median (IQR)	12.05 (10.65–13.23)
Exercise duration, median (IQR)	4 (4–6)
Surgical indications	
Adenocarcinoma	10
Bronchiectasis	8
Aspergilloma	7
Mucormycosis	6
Fibrocavitary disease	6
Carcinoid	4
Squamous	1
Mucoepidermoid	1
Leiomyosarcoma	1
Neuroendocrine	1
Hydatid cyst	1
Comorbidities	
Single comorbidity	
Diabetes mellitus	7
Hypertension	7
Coronary artery disease	3
Chronic lung disease	4
Multiple comorbidity	
DM+HTN	3
DM+CAD	2
DM+Asthma	1
HTN+COPD	1

Among the 46 subjects, 41 (89.1%) underwent lobectomy and 5 (10.9%) underwent pneumonectomy. Right-sided procedures were performed in 26 (56.5%) subjects and left-sided procedures in 20 (43.5%) subjects. Among the pneumonectomies, 2 (4.3%) subjects underwent right pneumonectomy and 3 (6.5%) subjects underwent left pneumonectomy.

QoL

QoL was assessed using the EQ-5D index before performing preoperative cardiopulmonary exercise testing and at the time of discharge. The EQ-5D index showed a significant improvement at discharge compared with preoperative QoL. Comparison using the Wilcoxon signed-rank test demonstrated a statistically significant difference between the two time points (*P* = 0.005). Comparison of QoL is shown in Table 2.

**Table 2:** Quality of life before surgery and at discharge. EQ-5D, EuroQol 5-Dimension questionnaire

Variable	Before surgery, median (Q1,Q3)	At discharge, median (Q1,Q3)	Test statistics (*Z*)	*P*-value
EQ-5D index	0.883 (0.796, 0.883)	0.883 (0.883, 0.883)	-2.83	0.005

Outcome variables

Postoperative Respiratory Complications

Among 46 patients, four (8.7%) developed postoperative respiratory complications; one (2.1%) had pneumonia with type 1 respiratory failure, and three (6.5%) subjects had pleural effusion. Postoperative respiratory complications were observed in one of five subjects (20%) who underwent pneumonectomy and three of 41 subjects (7.3%) who underwent lobectomy. Among the four subjects who developed postoperative respiratory complications, mucormycosis was present in three subjects (75%), while bronchiectasis was present in one subject (25%). The distribution of respiratory complications is shown in Table 3. 

**Table 3:** Association of preoperative VO₂max with postoperative complications. VO2max, maximum oxygen uptake

Variable	Value
EQ-5D before surgery (Median(Q1,Q3)	0.883 ((0.796,0.883)
EQ-5D at discharge(Median(Q1,Q3)	0.883 (0.883,0.883)
Z statistic	2.83
P value	P value
Pneumonia with respiratory failure	1 (2.17%)
Pleural effusion	3 (6.52%)
No complications	42 (91.3%)

Factors Associated With Complications

The mean preoperative FEV1 was compared between patients with and without postoperative complications and was found to be not statistically significant (1.67 ± 0.31 vs. 2.06 ± 0.58, *P* = 0.193).

The distribution of preoperative maximum oxygen consumption (VO₂max) was analyzed between subjects who developed postoperative respiratory complications and those who did not. 

The median (interquartile range (IQR)) VO₂max in patients without complications was 16.34 mL/kg/minute (15.12-17.37), whereas patients with complications had a median VO₂max of 8.91 mL/kg/minute (8.07-12.04), as shown in Table 4.

**Table 4:** Comparison of VO₂max and complications. VO_2_max, maximum oxygen uptake

Variable	No complications (*n* = 42), median (Q1,Q3)	Complications (*n *= 4), median (Q1,Q3)	Test statistics	*P*-value
VO₂max (mL/kg/minute)	16.34 (15.12, 17.37)	8.91 (8.07, 12.04)	Mann–Whitney U = 5.0	0.002*

On comparison using the Mann-Whitney U test, this difference was found to be statistically significant (P = 0.002), indicating that lower VO₂max values were associated with a higher occurrence of postoperative respiratory complications.

Logistic Regression Analysis for the Prediction of Postoperative Respiratory Complications

A univariable binary logistic regression analysis was performed to assess the association between VO₂max (continuous variable) and the occurrence of postoperative respiratory complications. VO₂max was found to be a significant predictor of postoperative respiratory complications. For each unit increase in VO₂max, the odds of developing postoperative respiratory complications decreased significantly (odds ratio (OR) = 0.58; 95% confidence interval (CI): 0.39-0.85; *P* = 0.005). This suggests that higher preoperative exercise capacity, as reflected by VO₂max, is associated with a lower risk of postoperative respiratory complications. Logistic regression analysis is shown in Table 5. 

**Table 5:** Predictive ability of VO₂max. VO_2_max, maximum oxygen uptake; CI, confidence interval

Predictor	Regression coefficient (β)	Standard error	Wald χ²	Odds ratio (95% CI)	*P*-value
VO₂max (mL/kg/minute)	-0.553	0.199	7.73	0.58 (0.39-0.85)	0.005*

*VO₂max and Mortality* 

To assess the association between preoperative exercise capacity and mortality, maximum oxygen consumption (VO₂max) was compared between survivors and non-survivors within 90 days after surgery. As VO₂max was a non-normally distributed continuous variable, comparison was performed using the Mann-Whitney U test. Patients who died within 90 days had a substantially lower VO₂max compared with survivors. The median (Q1, Q3) VO₂max among survivors was 16.21 (15.12, 17.22) mL/kg/minute, whereas among non-survivors, it was 7.8 (5.00, 7.80) mL/kg/minute. This difference was found to be statistically significant by the Mann-Whitney U test (*P* = 0.007), indicating that lower preoperative VO₂max was associated with increased mortality within 90 days after lung resection surgery. The association is shown in Table 6.

**Table 6:** Association of preoperative VO₂max with 90-day mortality (n = 46). VO₂max, maximum oxygen uptake

Variable	Survivors (*n* = 43), median (Q1,Q3)	Non-survivors (*n* = 3), median (Q1,Q3)	Test statistics	*P*-value
VO₂max (mL/kg/minute)	16.21 (15.12, 17.22)	7.80 (5.00, 7.80)	Mann-Whitney U = 4.0	0.007*

## Discussion

In the present study, the mean age of patients who underwent lung resection was 46.67 ± 14 years, indicating a relatively younger cohort compared with many lung resection studies [5]. The lower mean age observed in the present study may be attributed to the inclusion of patients with both malignant and non-malignant etiologies.

Although increasing age is regarded as a risk factor for adverse postoperative outcomes, some authors have observed that physiological parameters such as FEV₁, DLCO, and VO₂max are more reliable predictors of postoperative outcomes than demographic factors alone [6,7]. These findings support the concept that physiological reserve, rather than chronological age, should form the basis of preoperative risk assessment in patients undergoing lung resection.

In the present study, male subjects who underwent lung resection constituted 60.9% of the study population. A similar predominance has been reported in studies by Liu et al. and Wang et al. [6,7].

Differences in health-seeking behavior and willingness to undergo major surgical procedures might have also contributed to the observed gender distribution, although these factors were not evaluated in the present study.

In the present study, patterns of comorbidity were analyzed; 18 (39.1%) subjects had no associated comorbidity, whereas 28 (60.9%) had associated comorbidities. Seven (15.2%) subjects had multiple comorbidities. The presence of single and multiple comorbid conditions in this cohort indicates that several subjects had systemic illnesses that could have potentially influenced overall cardiopulmonary reserve.

There was no change in quality of life assessed using the EQ-5D index preoperatively before cardiopulmonary exercise testing and postoperatively at the time of discharge. The median EQ-5D score at presentation was 0.883. Despite the similarity in median values, paired analysis demonstrated a statistically significant improvement in scores at the time of discharge.

Since many patients already had relatively high baseline EQ-5D scores, the overall median remained unchanged, indicating a possible ceiling effect. In such situations, even modest improvements in individual patients may not shift the central value but can still be detected statistically. Early (<14 days) postoperative symptom relief, effective pain control, and supervised inpatient monitoring might have contributed to patient-reported QoL.

FEV₁ was not statistically significant when compared between subjects with complications and those without complications. Benzo et al. and Ferguson et al. have reported that spirometric parameters such as FEV₁ are useful in the preoperative assessment of lung resection candidates, although their predictive value may be limited when used alone [8,9].

Preoperative VO₂max showed a significant association with postoperative respiratory complications, with markedly lower values observed in subjects who developed complications. These findings indicate that reduced exercise capacity was linked to an increased risk of postoperative respiratory complications. Similar observations have been reported across multiple studies evaluating the role of cardiopulmonary exercise testing before lung resection. Benzo et al. demonstrated that lower VO₂max values were associated with increased postoperative morbidity and mortality [8]. Liu et al. and Mazzella et al. also reported higher rates of postoperative complications in patients with reduced preoperative exercise capacity (VO₂max) [6,10]. The systematic review by Arbee-Kalidas et al. reported consistent associations between lower VO₂-based parameters and adverse postoperative outcomes across large surgical cohorts [3]. Using a cutoff of VO₂max >15 mL/kg/minute, lower complication rates were observed in these studies. Subjects with lower VO₂max may have limited ability to meet the increased metabolic demands following lung resection, which can predispose them to secretion retention, ineffective cough, and postoperative respiratory complications.

In the present study, mortality was assessed during hospital stay and on the 7th, 30th, and 90th days through telephonic contact. The 90-day postoperative mortality was 6.5% (3 out of 46 subjects). Subjects who died had markedly lower preoperative VO₂max values compared with survivors, indicating that reduced preoperative exercise capacity was associated with higher early-term (14 days) postoperative mortality.

Rosen et al. reported a 90-day mortality of approximately 3.7% after pulmonary resection (lobectomy, pneumonectomy, and wedge resection), which was notably higher than the 30-day mortality rate [11].

Mazzella et al. demonstrated that lower exercise oxygen consumption was associated with increased postoperative mortality (90-day mortality of 6.7%) following lung resection [10]. These observations are consistent with the present study, where subjects who died had substantially lower VO₂max values than survivors.

In a surgical cohort by Skrzypczak et al. evaluating postoperative outcomes after lung resection, the 30-day and 90-day mortality rates were reported as 4.3% and 9.1%, respectively, with factors such as bronchopleural fistula, positive bronchial margin, chest wall infiltration, and prolonged intubation identified as significant predictors of mortality [12].

Although the number of deaths in the present study was small, the clear separation in VO₂max values between survivors and non-survivors suggests that reduced exercise capacity may help identify subjects at increased risk of adverse postoperative outcomes. These findings support the role of cardiopulmonary exercise testing as part of the preoperative assessment in subjects undergoing lung resection.

Limitations

Our study has the following limitations. The relatively small sample size may limit validation in an independent cohort, which may reduce statistical power and limit the generalizability of the findings. Larger, multicenter studies are needed to confirm these results. Additionally, the study included both malignant and non-malignant conditions for lung resection, which might have introduced variability in baseline characteristics and postoperative risk. Postoperative respiratory complications were evaluated during the early (<14 days) postoperative period, and late postoperative events (>90 days) were not assessed. The low number of postoperative complication events may limit the stability of the logistic regression estimates.

## Conclusions

Preoperative VO₂max measured by cardiopulmonary exercise testing was significantly associated with postoperative respiratory complications following lung resection surgery. Lower VO₂max independently predicted postoperative respiratory complications and was also associated with increased 90-day mortality. These findings suggest that incorporating VO₂max into the preoperative assessment may improve risk stratification and help identify patients at higher risk of adverse postoperative outcomes.
